# Socioeconomic Status, Reserve Capacity, and Depressive Symptoms Predict Pain in Rheumatoid Arthritis: An Examination of the Reserve Capacity Model

**DOI:** 10.21203/rs.3.rs-2758092/v1

**Published:** 2023-04-07

**Authors:** Desiree Azizoddin, Richard Olmstead, Kris-Ann Anderson, Alanna E. Hirz, Michael R. Irwin, Shadi Gholizadeh, Michael Weisman, Mariko Ishimori, Daniel Wallace, Perry Nicassio

**Affiliations:** University of Oklahoma Health Sciences Center; UCLA Geffen School of Medicine; University of Oklahoma Health Sciences Center; UCLA Fielding School of Public Health; UCLA Geffen School of Medicine; San Diego State University/University of California; Cedars-Sinai Medical Center; Cedars-Sinai Medical Center; Cedars-Sinai Medical Center; Cedars-Sinai Medical Center

**Keywords:** Rheumatoid arthritis, Pain, Socioeconomic factors, Psychosocial factors, Depressive symptoms

## Abstract

**Background:**

Guided by the reserve capacity model, we examined the roles of socioeconomic status (SES), reserve capacity, and negative emotions as determinants of pain in patients with Rheumatoid Arthritis (RA).

**Methods:**

The study used cross-sectional baseline data from 106 adults in a clinical trial comparing behavioral treatments for RA. Structural equation modeling evaluated the direct effects of SES, reserve capacity (helplessness, self-efficacy, social support) and negative emotions (stress and depressive symptoms) on pain, and the indirect effects of SES as mediated by reserve capacity and negative emotions.

**Results:**

Results showed that low SES contributed to greater pain, through lower reserve capacity and higher negative emotions. Mediational analyses showed that reserve capacity and negative emotions partially mediated the effect of SES on pain.

**Conclusions:**

The findings indicate that interventions that target negative emotions in patients with low SES may facilitate better pain control with RA.

**Trial registration:**

clinicaltrials.gov NCT00072657; 02/2004

## Background

Approximately 1.3 million adults in the United States have rheumatoid arthritis (RA), which is a debilitating autoimmune, systemic and chronic condition characterized by inflammation (1–3). Over time, RA affects larger joints and bodily organs and patients become at a heightened risk for developing pain, in addition to comorbid medical (e.g. heart disease) and psychiatric conditions (e.g. depression). Together these symptoms further exacerbate detriments in an individual’s quality of life, disability status/ability to work, and even early death (4). Pain, resulting from increased inflammation in joints, is widely common among RA and autoimmune populations and has been shown to significantly contribute to worse quality of life. Depressive psychological symptoms are also reported in 10–46% of individuals diagnosed with RA and is associated with increased inflammatory reactivity, significant pain, increased fatigue, increased disability, and lower quality of life (5–9). In addition to an exacerbation of disease-specific symptoms, patients with rheumatic diseases experiencing depressive symptoms and pain have also been shown to have poorer treatment adherence, increased emergency room visits, and higher medical costs relative to those without depressive symptoms (10).

No single cause of RA has been identified or implicated to solely drive patient outcomes but rather an aggregate of biological, social, environmental, and psychological factors appears to influence the course of the condition. However, recent efforts to appreciate the disparities in patient outcomes have demonstrated the unique role of socioeconomic status (SES). SES includes a composite of individual income, the amount and type of education received, occupational prestige, and status in a hierarchical society (11, 12). Socioeconomic determinants have been implicated in both the risk and trajectory of the condition, with those of lower SES having worse disease activity, mental and physical health, and quality of life relative to patients from higher SES (13–16). Past research has identified a relationship between SES and clinical outcomes in inflammatory diseases, specifically RA and lupus (17, 18).

While both socioeconomic status and depressive symptoms have been shown to render patients with RA to poorer disease activity and overall health comes, there is a paucity of literature examining the psychosocial mechanisms of these associations, particularly in the context of internal and external psychosocial resources with pain, a primary clinical symptom of RA. The mechanisms through which SES affects health outcomes in rheumatic disease are less clear suggesting a need for greater precision in how RA is conceptualized, especially as it relates to the role of psychosocial and socioeconomic factors and identifying appropriate intervention targets. Improved understanding of the unique contributions and pathways of internal resources (self-efficacy), psychosocial factors, and parameters of disease symptomatology could inform future research of rheumatic diseases through greater precision of conceptualizations of disparate health outcomes and future interventions.

The reserve capacity model is a framework for examining how over the long-term SES may affect health disparities and overall differences in health outcomes (19). Specifically, SES, above-and-beyond the influence of race, has been shown to have a gradient influence on health outcomes by exerting both direct and indirect effects on internal resources, which in turn, contribute to perceived stress and negative emotions. Stress and negative emotions are proposed to result in increases in patients’ inflammatory responses and consequently worse patient outcomes (e.g., greater inflammation related to increased pain symptoms or greater neurocognitive inflammation and worse psychological health). Accordingly, the reserve capacity model posits that lower SES may lead to more individually-experienced environmental and social stressors, which over time, may reduce or deplete internal (e.g., internal locus of control, optimism, hopefulness) and external (e.g., social support) psychosocial resources that may serve as protective factors in decreasing the effects of chronic stressors. While internal and external resources exist on a continuum, the lack of adequate resources to respond to the increased daily stressors diminishes the capacity of individuals to cope with stressors effectively, and ultimately contributes two-fold to health outcomes directly and indirectly. These effects may occur through increased autoimmune reactivity, negative emotions, negative behaviors, and persistent physiological arousal instability (20–22). The reserve capacity framework posits that the aggregate of resource factors, not single-factors of personal resources explain the relationship between SES and health, which has not yet been assessed in pain outcomes in RA – an autoimmune disorder often resulting in disabling pain and direct susceptibility to increased stress reactivity(23).

**Figure 1:**
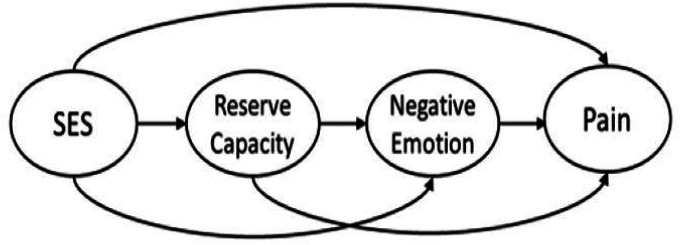
Hypothesized direct and indirect effects of SES, reserve capacity, and negative emotions on Rheumatoid Arthritis pain.

Applying the reserve capacity model in adults diagnosed with RA, we sought to evaluate predictors of pain intensity, one of the most important patient-reported outcomes in rheumatic disease. In application, the reserve capacity model could shed light on modifiable factors that may serve as targets for interventions to possibly improve health disparities, and decrease pain and other negative health outcomes in patients with RA. Specifically, our primary objective was to evaluate a model proposing that SES would contribute to subjective pain intensity directly, and indirectly through the potential mediators of reserve capacity and negative emotions (see Fig. 1). We hypothesized that: (1) higher SES would be positively associated with reserve capacity; (2) higher reserve capacity would be related to lower levels of negative emotions; (3) lower levels of negative emotions would be related to lower pain intensity; (4) reserve capacity would mediate the relationship between SES and pain; and, (5) negative emotions would mediate the relationship between SES and pain.

## Materials And Methods

### Participants and Procedures

This study used cross-sectional baseline data from adults with RA from the greater Southern California area who participated in a clinical trial that compared behavioral treatments for RA. This study received approval from the Institutional Review Board at the study site. Participants were recruited from clinic offices in the divisions of rheumatology at an academic medical center and a private hospital system and through newspaper advertisements and flyers posted in the community. After obtaining participants’ informed consent, the study rheumatologist at completed a physical examination during which the diagnosis of RA was confirmed. Participants were then referred to the academic medical center for an evaluation of their clinical functioning and psychiatric status. Reports of medication use were also obtained, including analgesics/nonsteroidal anti-inflammatory drugs, biologic agents, disease-modifying anti-rheumatic drugs (DMARDs), and “other” medication (drugs for other medical conditions, including psychotropic agents). Details on the participant recruitment and evaluation process have been reported previously (24).

Eligible participants had to (a) be 18 years of age or older, (b) meet the American College of Rheumatology revised criteria for RA, (c) have a stable disease-modifying drug regimen for 3 months prior to study entry,(d) have a stable disease course for 3 months, (e) be free of serious co-morbid medical conditions such as diabetes, congestive heart failure, renal failure, cancer, or fibromyalgia, that would confound interpretations of health status, (f) not be pregnant, (g) not have a serious psychiatric condition such as bipolar disorder, psychosis, or post-traumatic stress disorder, (h) not be suicidal, and (i) not have previous experience with cognitive behavioral therapy. All participants underwent a psychiatric evaluation using the Structured Clinical Interview for DSM Disorders, (25) under the direction of the project psychologist and psychiatrist.

### Measures

The structural model evaluated in this study (Fig. 1) was comprised of the constructs of SES, reserve capacity, negative emotions, and pain. Educational attainment and household income were used as indicators of the latent variable, socioeconomic status. Specifically, participants were asked to indicate their number of years of education and their total annual household income.

Identified as a limitation in research demonstrating the mediators of the relationship between SES and health outcomes, our analyses distinguished reserve capacity mediators as the aggregate of resilience factors that have been previously identified as comprising aspects of reserve capacity (20, 23). Reserve capacity was included as a latent variable with 3 indicators representing the Personal Mastery Scale (PMS;(26)), the Social Provisions Scale (SPS; (27)), and the Arthritis Helplessness Index (AHI; (28)). The PMS is a 7-item scale that measures the extent to which an individual perceives a sense of optimism, personal control, or mastery over life outcomes. Responses are measured on a scale and total scores may range from 7–28, with higher scores reflecting a greater sense of personal mastery. The SPS assesses 6 functions or “provisions” that may be derived from social relationships (i.e., attachment, social integration, opportunity for nurturance, reassurance of worth, reliable alliance, and guidance). Items are rated on a 4-point scale; the total sum score may range from 24 to 96, with higher scores signifying greater perceived social support. The AHI is a 15-item questionnaire designed to measure participants’ perceptions helplessness and loss of control focusing on a patient’s external, health-related locus of control in association with their arthritis symptoms and pain. Items are rated on a 6-point scale and the sum score may range from 15 to 90. For analysis purposes, AHI items were reverse scored so that higher scores on this measure reflected less external, health-related locus of control and helplessness, and therefore higher levels of reserve capacity (19).

Negative emotion was included as a latent variable with 3 indicators representing the Perceived Stress Scale (PSS; (29)), the Hamilton Depression Rating Scale (HDRS; (30, 31)) and the Negative Affect Scale of the Positive Negative and Affect Schedule (PANAS; (32)). The PSS is a 10-item scale that assesses the degree to which participants find their lives to be unpredictable, uncontrollable, and overwhelming. Responses are measured on a 4-point scale; the sum score may range from 0 to 40, with higher scores indicating greater perceived stress. The HDRS is an observer-rated assessment of the nature and severity of mood, anxiety, neurovegetative, and cognitive symptoms associated with depression. The 17 items are rated on a 0–4 or 0–2 scale, and total scores may range from 0 to 50, with higher scores signifying the presence of more severe depressive symptoms. A trained project research assistant completed the HDRS on each research participant. The Negative Affect Scale of the PANAS contains a list of 10 mood adjectives and measures the extent to which participants experience negative affective states (e.g., anger, guilt, and nervousness). Items are rated on a 5-point scale, and higher scores represent greater subjective distress and negative affectivity.

Two indicators were used to measure the latent variable, arthritis pain: the total joint score from the Rapid Assessment of Disease Activity in Rheumatology (RADAR; (33)) and a pain visual analogue scale (VAS). The RADAR total joint score assess joint pain/tenderness in 10 joints on the right and left sides of the body. Items are rated on a 4-point scale and total scores may range from 0 to 60, with higher scores indicating more severe joint pain. On the pain VAS, participants indicated the severity of their arthritis pain by placing a mark on a 10.0 cm horizontal line anchored by no pain (0 cm) and severe pain (10.0 cm). The pain VAS score measured the distance from the scale origin (0 cm) to point on the line marked by the participant.

### Data Analyses

Structural equation modeling (SEM) was used to examine the proposed model and analyses were conducted using EQS 6.1 (34). The associations between medication use (i.e., analgesics/nonsteroidal anti-inflammatory drugs, biologic agents, DMARDs, and other medications) and the model indicator variables were assessed to determine their potential impact on model findings. If statistically significant, covariates of medication use related to RA disease and pain were partitioned from relevant indicators prior to analyses. The rule of a minimum of 10 cases to the number of measured variables was used in determining the adequacy of the data for testing the model. Additionally, > 80% power for the regression coefficients among latent variables in the model required 85 cases. The SEM model was evaluated using multiple fit criteria: χ^2^ goodness-of-fit statistic, the comparative fit index (CFI), the standardized root mean square residual (SRMR), and the root mean square error of approximation (RMSEA). A statistically nonsignificant χ^2^ (p > .05) is suggestive of a good match between the data and the hypothesized model. A CFI value greater than .95 is considered evidence of a good fitting model (35). For SRMR, a value < .08 is considered acceptable (36). A RMSEA < .08 may also be indicative of good fit (37). Model modifications were performed based on results from the Wald test and Lagrange multiplier (LM) test, along with theoretical considerations.

Mediation analyses examined the extent to which reserve capacity and negative emotions mediated the effect of SES on pain. First, the preconditions for mediation were assessed to confirm that SES was significantly related to pain and the hypothesized mediators (i.e., reserve capacity and negative emotions)(38). Then, single mediator models were assessed to discern the mediating effects of reserve capacity and negative emotions separately. The 3-path mediated effect was also examined (i.e. the indirect effect from SES to pain mediated by reserve capacity and negative emotions). Statistical significance of the indirect effect, reflective of a significant decrease in the direct influence of SES on pain, was taken as evidence of mediation (38). The significance of indirect effect estimates was calculated by EQS, based on the Sobel method (39). Elimination of the initially significant direct effect of SES on pain indicated full mediation; partial mediation was established if the strength of this association was diminished but still significant(38). The 3-path mediated effect was also evaluated with the joint significance test, which offers evidence of mediation provided all paths involved in the collective indirect effect are significantly non-zero (40).

## Results

A total of 106 participants were included in the study. Table 1 shows demographic characteristics of the participants. The sample consisted of 90 females and 16 males, with an average age of 55.45 years and illness duration of 10.63 years. Participants came from a range of ethnicities. Whites were the most prevalent group, but participants from African-American, Hispanic, and Asian ethnicities were also represented. The sample can be characterized as middle to upper-middle class, possessing almost 16 years of education on average, and an annual income of greater than $50,000.

### Preliminary Analyses

Prior to testing the model, the data were screened, and results revealed a normal distribution and no multivariate outliers. Table 2 includes the intercorrelations, means and standard deviations for all observed variables represented in the structural model. Evaluation of the relationships among the latent constructs indicated moderate to strong associations between SES and pain (*r* = − .563, *p* < .001), and between SES and the 2 posited mediators (for reserve capacity: *r* = .513, *p* < .001; for negative emotions: *r* = − .363, *p* < .001), con rming that the preconditions for mediation were present. In the assessment of covariates, use of DMARD medications was significantly associated with RADAR total joint score (*r* = − .334, *p* < .001), pain VAS, (*r* = − .287, *p* = .002), PANAS negative affect scale (*r* = − .232, *p* = .014), and HDRS (*r* = − .324, *p* < .001). The variance explained by this covariate was partitioned from the noted indicators prior to structural equation modeling analyses.

### SEM Results

**Figure 2:**
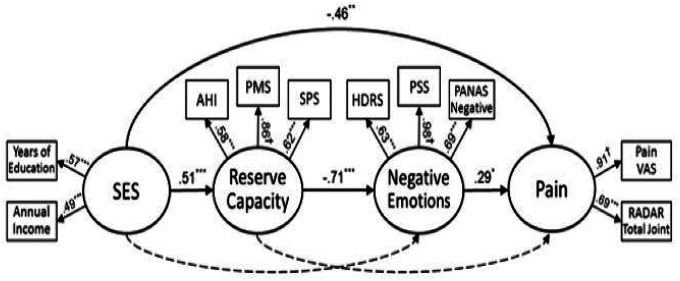
Final structural model with standardized path coefficients and factor loadings of SES, reserve capacity, and negative emotions on Rheumatoid Arthritis pain. Notes. ^†^pathway set to 1.0. Dashes lines indicate paths dropped from model. AHI = Arthritis Helplessness Index. PMS = Personal Mastery Scale. SPS = Social Provisions Scale. HDRS = Hamilton Depression Rating Scale. PSS = Perceived Stress Scale. PANAS Negative = Negative Affect scale of the Positive and Negative Affect Schedule. RADAR Total Joint = Rapid Assessment of Disease Activity in Rheumatology total joint score. Pain VAS = Pain visual analog scale. *p<.05; **p<.01; ***p<.001.

The hypothesized model (as depicted in Fig. 1) provided only a marginal fit to the observed data, CFI = .937; χ^2^(29) = 51.14, *p* = .007; SRMR = .061; RMSEA = .085. Post hoc modifications were performed using the LM and Wald tests in an attempt to develop a better fitting and simpler model. Based on the LM test and theoretical plausibility, the error terms for HDRS and PANAS negative affect were covaried (*cov* = .497, p < .001), which resulted in an improvement in model fit [CFI = .982; χ^2^(28) = 34.20, *p* = .194.; SRMR = .051; RMSEA = .046]. However, the Wald test indicated that the impact of deleting the non-significant paths from SES to negative emotions (β = .082, *p* = .616) and from reserve capacity to pain (β = .031, *p* = .905) on the χ^2^ of the model would be minimal. As such, in an effort to attain parsimony, these paths were removed. The fit of this revised model was similar: CFI = .987; χ^2^(30) = 34.42, *p* = .264; SRMR = .051; RMSEA = .037, and the model now consisted of only statistically significant paths (p < .05; Table 3). The LM test and the Wald test did not indicate any further improvement of the model through the addition or deletion of paths. Overall, the specified predictors explained 26% of the variance in reserve capacity, 50% of the variance in negative emotions, and 39% of the variance in pain. The final model with standardized path coefficients is shown in Fig. 2.

The final model of relations among SES, reserve capacity, negative emotions, and pain fit the data well. Inspection of the path coefficients showed that SES exerted a direct and negative effect on pain (β = − .46, *p* = .004). Furthermore, SES directly and positively related to reserve capacity (β = .51, *p* < .001), and greater reserve capacity predicted lower levels of negative emotions (β = − .71, *p* < .001). In turn, negative emotions had a direct and positive effect on pain intensity (β = .29, *p* = .024).

### Mediation Analyses

In the single mediator models, a direct relationship was specified between SES and pain, and an indirect (mediating) effect though either reserve capacity or negative emotions. The 2 single mediator models demonstrated adequate t [CFI = .951; χ^2^(11) = 18.56, *p* = .069; SRMR = .050; RMSEA = .080 for reserve capacity; CFI = 1.00; χ^2^(10) = 8.69, *p* = .562; SRMR = .041; RMSEA < .001 for negative emotions]. SES was predictive of reserve capacity (β = .54, *p* = .002); however, the effect of reserve capacity on pain did not reach statistical significance (β = − .22, *p* = .210). Thus, reserve capacity did not mediate the relationship between SES and pain (βindirect = − .12, *p* = .165). While SES also had a direct effect on negative emotions (β = − .35, *p* = .025), negative emotions were predictive of pain (β = .31, *p* = .032), indicating negative emotions mediated the association between SES and pain (β_indirect_ = − .11, *p* = .048). This finding, in combination with the attenuated but still significant direct effect of SES on pain, suggests that the association between SES and pain was partially mediated by negative emotions.

Finally, the 3-path mediated effect from SES to pain was evaluated. A test of the 3-path mediated effect of the sequence of processes depicted in Fig. 2 supported that the collective indirect effect—from SES to pain mediated by reserve capacity and negative emotions serially—was statistically explicated through the 2 mediational variables (βindirect = − .10, *p* = .029). The joint significance test also evidenced mediation because each of the 3 paths in the collective indirect effect was significant (40). Since the direct effect of SES on pain remained significant after controlling for the 2 mediational variables, the association between SES and pain was partially mediated by reserve capacity and negative emotions.

## Discussion

This study sought to evaluate the role of SES, reserve capacity, and psychological factors on pain outcomes, a highly relevant clinical symptom in patients with RA. In this sample of mostly married, white, female patients with physician-diagnosed RA, after controlling for relevant medication use, the present study demonstrated that socioeconomic status was significantly associated with pain intensity. Additionally, patients’ levels of reserve capacity including their reported helplessness, self-efficacy, and social support (see Fig. 2) and negative emotions significantly mediated this relationship between SES and pain intensity. Our findings are consistent with prior studies in rheumatologic clinical cohorts – SES was related to worse clinical outcomes including pain (41–43). Negative emotions and reserve capacity (helpless, social support and self-efficacy) mediated the effect of SES on pain, above and beyond the impact of patient’s RA medications, together explaining 39% of the variance in pain intensity.

Although reserve capacity was not shown to independently mediate the SES–pain relationship, results indicated that it was inversely related to negative emotions, a direct mediator of the relationship between SES and pain. Taken together, these data suggest that negative emotions result in worse pain and that high reserve capacity functions as a protective factor against negative emotions and contributes to lower pain intensity indirectly. The findings provide evidence that lower reserve capacity (higher helplessness, lower self-efficacy, and lower social support) to manage internal and external stressors results in increased negative emotions, which play a critical and unique role in explaining how lower SES results in increased pain for patients with autoimmune disorders such as RA. In turn, higher SES can be associated with fewer and less severe environmental/social stressors and increased psychosocial reserve capacity resources (financial resources, social support, optimism, and self-esteem) to respond to these stressors, therefore leading to less inflammatory reactivity and negative emotions, that can then result in improved health outcomes. Prior studies evaluating a range of medical diagnoses have shown that reserve capacity resources are related to improved emotional adjustment with lower negative emotions, potentially through reduced inflammatory reactivity in the CNS, and subsequently more positive health functioning.(20, 22, 44–49). This study established the relevance of the reserve capacity framework in explaining pain outcomes in RA. These findings help highlight the value of an integrated, theoretical framework that enables examination of potential underlying mechanisms to understand the pain specific inequities that result for patients with lower SES and to help identify future intervention targets.

SES, while highly related to clinical outcomes in RA, is di cult, if not impossible, to modify. These data support results from prior studies that show negative emotions and reserve capacity resources are both related to rheumatologic outcomes and are *modifiable factors* that can be the focus of behavioral interventions to facilitate better control of pain and other clinical outcomes in patients with RA (11, 50–54). Comparable findings among SLE cohorts further underscore the clinical value of behavioral interventions in addressing the indirect effect lower SES may have on disease-related morbidity (42, 55). Our findings also strengthen the path for future research that aims for greater precision in understanding how SES determinants and psychosocial factors contribute to the experience of rheumatologic conditions and patient-reported outcomes. Results support the testing of and evidence for the reserve capacity model as a model that may have utility for studying and treating rheumatologic populations more equitably. Future research is needed that can examine the effects of racial inequities, SES, negative emotions, and reserve capacity on additional parameters of disease processes in RA. More specifically, future intervention research should evaluate the potential impact of psychosocial interventions that target coping with negative emotions on relevant patient reported outcomes in RA including pain.

This study has many strengths as a novel comprehensive evaluation of biological, socioeconomic, psychological, and social factors on self-reported and clinician evaluated pain in RA. The study sample was also validated further as patients were confirmed to have RA by physician evaluation. The study also has limitations. Although the sample was racially diverse, the majority of the patients in this sample were highly educated and above the poverty line – education and income can play an equally important role as social determinants of health (14–16, 42). Yet, the mean income of participants recruited to the study was $50,262 ± 15,593, which was slightly above the median per capita income in the U.S. at that time - $47.828, making the sample similar in representation of the general U.S. public financially. Of particular note, however, participants were recruited from Los Angeles county, the 8th most expensive city in the world (during the recruitment period) which likely influences patients’ stress related to financial well-being. Even still, this study identified worse pain outcomes for those with lower SES. The findings can only be generalized to similar samples and future studies are needed to evaluate the mediational role of negative emotions and reserve capacity on SES and pain in larger samples with greater racial, income, and educational diversity. Lastly, the study utilized cross-sectional data which may limit the interpretability of the influence of symptoms on each other over time within the reserve capacity model. Future studies would benefit from using longitudinal designs to determine the directionality and impact of these factors on pain outcomes in RA.

## Figures and Tables

**Table 1:** Demographic characteristics of sample (n = 106)

	Mean ± SD or *n* (%)		Range	
Age in years	55.45	± 12.02	22	−79
Education in years	15.90	± 2.29	12	−21
Annual income ($)	50,262	± 15,593	23,910	−112,521
Female	90	(84.91)		
Race/Ethnicity				
White	64	(60.38)		
Hispanic	17	(16.04)		
Black	14	(13.21)		
Asian/Pacific Islander	9	(8.49)		
Other race/ethnicity	2	(1.89)		
Marital status				
Never married	15	(14.15)		
Divorced/Separated	25	(25.58)		
Widowed	10	(9.43)		
Married	56	(52.83)		
Years since RA diagnosis	10.63	± 10.75	1	−53

**Table 2:** Intercorrelations, means, and standard deviations for model variables

	1.	2.	3.	4.	5.	6.	7.	8.	9.	10.
1. Education (in years)	--									
2. Annual income ($)	.28**	--								
3. AHI total	.17	.14	--							
4. PMS total	.25**	.21**	.49***	--						
5. SPS total	.18	.15	.36***	.53***	--					
6. PSS total	− .20*	− .17	− .40***	− .59***	− .42**	--				
7. HDRS total	− .13	− .11	− .25**	− .38***	− .27**	.61***	--			
8. PANAS negative affect	− .14	− .12	− .28**	− .41***	.52***	.67***	.71***	--		
9. RADAR total joint	− .22*	− .18	− .17	− .25**	− .18	.30**	.19*	.21*	--	
10. Pain VAS	− .29**	− .25**	− .23*	− .34***	− .24*	.40***	.25**	.28**	.62***	--
*M*	15.90	50,262	52.89	23.90	84.26	11.14	4.34	14.18	12.13	3.01
*SD*	2.29	15,593	9.51	3.06	8.67	6.73	4.91	5.46	9.28	2.39
*a*	NA	NA	.41	.78	.79	.47	.79	.91	.91	NA

Notes. AHI = Arthritis Helplessness Index. PMS = Personal Mastery Scale. SPS = Social Provisions Scale. PSS = Perceived Stress Scale. HDRS = Hamilton Depression Rating Scale. PANAS = Positive and Negative Affect Schedule. RADAR = Rapid Assessment of Disease Activity in Rheumatology. VAS = Visual Analog Scale.

* p < .05;

** p < .01;

*** p < .001.

**Table 3:** Standardized direct, indirect and total effects from final model

	Direct	Indirect	Total
SES ◊ Reserve capacity	.51***	—	.51***
SES ◊ Negative emotion	—	− .36**	− .36**
SES ◊ Pain	− .46**	− .10*	− .56***
Reserve capacity ◊ Negative emotion	− .71***	—	− .71***
Reserve capacity ◊ Pain	—	− .20*	− .20*
Negative emotion ◊ Pain	.29*	—	.29*

* p < **.05**;

** p < **.01**;

*** p < **.001**.

## Data Availability

De-identified data from this study are not available in a public archive. De-identified data from this study will be made available (as allowable according to institutional IRB standards) by emailing the corresponding author. Materials used to conduct the study are not publicly available.

## Abbreviations

AHI: Arthritis Helplessness Index
CFI: Comparative fit index
DMARDs: Disease-modifying anti-rheumatic drugs
HDRS: Hamilton Depression Rating Scale
LM: Wald test and Lagrange multiplier test
PANAS: Negative Affect Scale of the Positive Negative and Affect Schedule
PMS: Personal Mastery Scale
PSS: Perceived Stress Scale
RA: Rheumatoid arthritis
RADAR: Rapid Assessment of Disease Activity in Rheumatology
RMSEA: Root mean square error of approximation
SEM: Structural equation modeling
SES: Socioeconomic status
SPS: Social Provisions Scale
SRMR: Standardized root mean square residual
VAS: Pain visual analogue scale
